# Practices for promoting sleep in intensive care units in Brazil: a national survey

**DOI:** 10.5935/0103-507X.20200043

**Published:** 2020

**Authors:** Fernando José da Silva Ramos, Leandro Utino Taniguchi, Luciano Cesar Pontes de Azevedo

**Affiliations:** 1 Unidade de Terapia Intensiva, Hospital Beneficência Portuguesa - BP Mirante - São Paulo (SP), Brasil.; 2 Unidade de Terapia Intensiva, Hospital Sírio-Libanês - São Paulo (SP), Brasil.; 3 Departamento de Emergência, Hospital das Clínicas, Faculdade de Medicina, Universidade de São Paulo - São Paulo (SP), Brasil.; 4 Brazilian Research in Intensive Care Network (BRICNet) - São Paulo (SP), Brasil.

**Keywords:** Sleep, Delirium, Patient-centered care, Surveys and questionnaires, Intensive care units, Brazil, Sono, Delirium, Assistência centrada no paciente, Inquéritos e questionários, Unidades de terapia intensiva, Brasil

## Abstract

**Objective:**

To conduct a national survey of intensive care professionals to identify the practices for promoting sleep in adult intensive care units in Brazil and describe the professionals’ perceptions of the importance of sleep for patients.

**Methods:**

An electronic questionnaire was distributed by the clinical research cooperation network of the *Associação de Medicina Intensiva Brasileira* and by the Brazilian Research in Intensive Care Network to physicians and nurses registered with the association. The questionnaire evaluated the profile of the respondents, the profile of their intensive care units, whether protocols for promoting sleep were present, the pharmacological and nonpharmacological measures typically employed in the unit, and the professionals’ perceptions regarding sleep in critically ill patients.

**Results:**

A total of 118 questionnaires were evaluated. The Southeast region of the country was the most represented (50 questionnaires, 42.4%). The majority of units had a clinical-surgical profile (93 questionnaires; 78.8%), and 26 had a continuous visitation policy (22.0%). Only 18 intensive care units (15.3%) reported having protocols for promoting sleep. The most cited measure for sleep promotion was reducing light during the night (95 questionnaires; 80.5%), which was more often performed in private intensive care units. Almost all of the responders (99%) believed that poor-quality sleep has a negative impact on patient recovery.

**Conclusion:**

The responses to this Brazilian survey revealed that few intensive care units had a program for promoting sleep, although almost all participants recognized the importance of sleep in patient recovery.

## Abstract (pt)

**Objetivo:**

Realizar um inquérito nacional com profissionais de terapia intensiva para determinar as práticas de promoção do sono em unidades de terapia intensiva para adultos no Brasil, e descrever suas percepções sobre a importância do sono para os pacientes.

**Métodos:**

Um questionário eletrônico foi distribuído pela rede de cooperação em pesquisa clínica da Associação de Medicina Intensiva Brasileira aos médicos e enfermeiros registrados na associação e pela *Brazilian Research in Intensive Care Network*. O questionário avaliou o perfil dos respondedores, de suas unidades de terapia intensiva, se estavam presentes protocolos de promoção do sono, quais as medidas farmacológicas e não farmacológicas usualmente empregadas na unidade e a percepção dos profissionais em relação ao sono nos pacientes críticos.

**Resultados:**

Foram avaliados 118 questionários. A Região Sudeste foi a mais representada (50 questionários; 42,4%). A maioria apresentava perfil clínico-cirúrgico (93 questionários; 78,8%) e 26 possuíam política de visita contínua (22,0%). Apenas 18 unidades de terapia intensiva (15,3%) referiram apresentar protocolos de promoção do sono. A medida mais citada para promoção de sono foi a redução da luminosidade no período noturno (95 questionários; 80,5%), sendo mais executada em unidades de terapia intensiva privadas. Quase a totalidade dos respondedores (99%) acreditou que o sono com qualidade ruim tinha impacto negativo na recuperação do paciente.

**Conclusão:**

Nas respostas deste inquérito brasileiro, poucas unidades apresentaram um programa de promoção de sono na unidade de terapia intensiva, embora a quase totalidade dos participantes reconhecesse a importância do sono na recuperação do paciente.

## INTRODUCTION

Sleep can be defined as a cyclical and reversible physiological process of disconnecting from the environment.^(1^) Sleep disorders (characterized by frequent interruptions, fragmentation, circadian rhythm disturbance, and loss of deep-sleep stages) are associated with unfavorable outcomes in critically ill patients.^(1-3^) Environmental factors that favor sleep disorders in intensive care units (ICU) include the noise of alarms and of activities of multidisciplinary team professionals, direct or indirect light, and manipulation of patients to measure vital signs, perform tests, and administer drugs.^(3,4^) In addition to the environment, the clinical condition and therapeutic interventions favor changes in sleep architecture in hospitalized patients (e.g., organ dysfunction, systemic inflammatory response, pain, stress, and medications such as vasopressors, antibiotics, sedatives, and analgesics).^(2,5^

Although a significant portion of health care professionals recognize the importance of sleep for critically ill patients, fewer than 30% of ICUs report having a program for promoting sleep.^(6^) A recent American College of Critical Care Medicine (ACCCM) guideline on pain, agitation/sedation, *delirium*, immobility, and sleep disruption (PADIS) recommends that sleep be promoted by optimizing the environment by reducing noise, light, and nocturnal stimuli to protect the sleep-wake cycle.^(7^) However, in Brazil, there is little information on practices for promoting sleep in the intensive care setting and how these recommendations are applied.

Thus, due to the scarcity of Brazilian data, the present study aimed to evaluate the main sleep promotion practices in adult ICUs in Brazil.

## METHODS

A national descriptive survey was conducted. Intensive care physicians and nurses with an electronic address listed in the research groups of the clinical research cooperation network of the *Associação de Medicina Intensiva Brasileira* (AMIBNet) and Brazilian Research in Intensive Care Network (BRICNet) were invited to participate in the study. A total of 1,622 electronic invitations were sent to medical professionals and 523 to nursing professionals from all Brazilian regions listed in AMIBNet. Additionally, an electronic invitation was sent to physicians and nurses with e-mails listed in a Yahoo^®^ group.

Study participants received an invitation to participate in the study and a link to the SurveyMonkey^®^ platform. A reminder to participate in the study was forwarded by AMIBNet to the same mailing lists after 1 month. The link remained valid from April 3, 2019 to June 10, 2019.

After agreeing with the participation conditions, the study participants were directed to fill out the electronic questionnaire (Appendix 1), which contained 44 questions on ICU identification, participant profile (profession and position), ICU structure (type, funding source, number of beds, type of division between beds), visitation policy (number of periods and length of each visit), nonpharmacological measures for promoting sleep (nocturnal noise reduction, light reduction, use of ear plugs or eye mask, sleep interruptions for tests, and/or radiography), and pharmacological measures for inducing sleep (melatonin, neuroleptics, benzodiazepines, and other drugs). The questionnaire exclusion criteria were as follows: not filling out the hospital name or city of the ICU, answering fewer than 50% of the questions, nonadult ICU, and duplicate ICU. In cases of duplicate responses from the same unit, only one questionnaire was considered valid, with the first choice being that from the physician coordinator, followed by the staff physician, nurse coordinator, on-duty physician, and on-duty nurse. Thus, each questionnaire analyzed represented a single ICU. The study was approved by the Research Ethics Committee of *Hospital Beneficência Portuguesa de São Paulo* under opinion number 3,238,465.

### Statistical analysis

The data collected were analyzed using the Statistical Package for Social Sciences (SPSS), version 20 (IBM, Illinois, USA). Categorical variables are described as absolute and relative frequencies. Quantitative variables are expressed as measures of central tendency (mean and median) and dispersion. To compare data on sleep practices between different kinds of ICU, the chi-squared test or Fisher’s exact tests was used for categorical data. Values of p < 0.05 (two-tailed) were considered significant.

## RESULTS

A total of 190 responses to the electronic survey were received. After assessing these questionnaires, 72 were excluded. The main causes of exclusion were not identifying the hospital or city of origin of the ICU and duplicate questionnaires from the same ICU. The other 118 questionnaires were considered valid and were included in the statistical analysis. Table 1 shows the profile of the units according to location. Among the study participants, 80.5% were physicians, and 29.7% of the sample identified themselves as physician coordinators of the unit. The results regarding the structural characteristics of the evaluated units are presented in table 2. Of the 118 evaluated ICUs, 96 (81.4%) reported having a sedation and analgesia protocol. The Richmond Agitation and Sedation Scale (RASS) was the most frequently used in the ICUs that had sedation and analgesia protocols (94 units). The main sedation strategy used in these ICUs was daily awakening (55 ICUs; 57.3%), and the intensive care physician was responsible for implementing the sedation protocol in 85 (88.5%) units. In the evaluated sample, only 18 units (15.3%) reported having a sleep promotion protocol.

**Table 1:** Profile of the intensive care units according to location

Region	Cities	Number of ICUs	Private ICUs per region
	(N)	N (%)	
Center-West	8	11 (9.3)	8 (72.7)
Northeast	12	26 (22.0)	8 (29.6)
North	5	8 (6.8)	4 (50)
Southeast	22	50 (42.4)	27 (54)
South	13	23 (19.5)	13 (56.5)

ICU - intensive care unit.

**Table 2:** Characteristics of the intensive care units

Characteristics	N (%)
Specialty	
Clinical	9 (7.6)
Surgical	1 (0.8)
Clinical-surgical (general)	93 (78.8)
Other (specialties)	15 (12.7)
Number of beds	
Up to 10	48 (40.7)
11 - 20	43 (36.4)
21 or more	27 (22.9)
Presence of a staff physician	
Yes	111 (94.1)
No	7 (5.9)
Nurse-patient ratio in the day	
1: ≤5	62(52.6)
1: 6 - 8	28 (23.7)
1: ≥ 9	28 (23.7)
Nurse-patient ration at night	
1: ≤ 5	32 (27.1)
1: 6 - 8	38 (32.2)
1: ≥ 9	48 (40.7)
Division between beds	
None	4 (3.4)
Partition	21 (17.8)
Curtain	48 (40.7)
Screen	6 (5.1)
Box/individual apartments	39 (33.1)
Has windows in most beds	
Yes	82 (69.5)
No	36 (30.5)
Has a clock near the beds	
Yes	65 (55.1)
No	53 (44.9)
Number of family visitation periods	
1	27 (22.9)
2	40 (33.9)
3	25 (21.2)
Continuous (24 hours)	26 (22.0)

**Figure 1:**
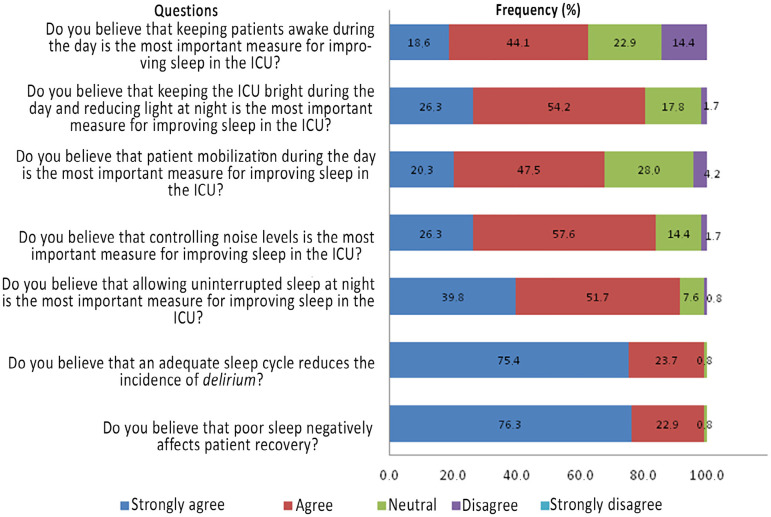
Participants’ perception of measures for promoting sleep in the intensive care unit. ICU - intensive care unit.

The main medications used among participants who reported having pharmacological sleep promotion protocols in the ICU were dexmedetomidine (11 units) and neuroleptics (10 units). The most widely used measure to promote sleep was reducing light at night, and the least performed measure was offering relaxing music to help induce sleep (Table 3). Figure 1 shows the perceptions of the study participants about the main sleep promotion measures in the ICU. Approximately 99% of the participants believed that poor-quality sleep in the ICU could negatively affect patient recovery. Table 4 shows the relationship between the funding source and sleep promotion measures in the ICU: there was no difference in the presence of sleep promotion protocols according to funding source. Reducing light at night was the only sleep promotion measure with a significant difference in prevalence between public and private ICUs (p = 0.01).

**Table 3:** Sleep promotion measures in intensive care units

Characteristic	N (%)
Nonpharmacological protocol for sleep promotion in the ICU	18 (15.3)
Measurement of noise in the ICU	17 (14.4)
Protocol for noise reduction in the ICU	17 (14.4)
Adjustment of alarm volumes at night	32 (27.1)
Light reduction at night	95 (80.5)
Minimize routine exams at night	61 (51.7)
Provides ear plugs to minimize noise	8 (6.8)
Provides eyes mask to reduce light	5 (4.2)
Provides music to help induce sleep	3 (2.5)
How do you assess patient sleep in the ICU?	
I do not routinely assess it	37 (31.4)
Report from multidisciplinary team	29 (24.6)
Report from patient	52 (44.1)
Does the ICU have a pharmacological protocol for promoting sleep?	
Yes	18 (15.3)
No	100 (84.7)
What is the approximate frequency of patients who received sleep promotion medication in the ICU in the last 7 days?	
Do not know	8 (6.8)
0 - 25%	32 (27.1)
26 - 50%	40 (33.9)
51 - 75%	29 (24.6)
76 - 100%	9 (7.6)

**Table 4:** Relationship between funding source and measures to promote sleep in intensive care units

	Private	Public	p value*
Nonpharmacological protocol for sleep promotion in the ICU			
Yes	10	8	0.799
No	50	50	
Protocol for noise reduction in the ICU			
Yes	11	6	0.29
No	49	52	
Reduces light at night			
Yes	54	41	0.01
No	6	17	
Minimizes routine exams at night			
Yes	32	29	0.85
No	28	29	
Pharmacological protocol for sleep promotion			
Yes	13	5	0.07
No	47	53	
Sedation and analgesia protocol			
Yes	50	46	0.64
No	10	12	

ICU - intensive care unit.

* Fisher's exact test with exact two-tailed significance.

## DISCUSSION

Our study, one of the first to evaluate practices for promoting sleep in Brazilian ICUs, has the following main findings: a small portion of the evaluated ICUs had a sleep promotion program; the most frequent measure for promoting sleep was reducing light at night; approximately 30% of the participants reported that they did not routinely assess the sleep of their patients, but 65% reported that more than half of their patients received sleep induction medication (dexmedetomidine being the main medication used); almost all the participants believed that poor sleep quality can negatively affect patient recovery and believed that adequate sleep reduces the incidence of *delirium*.

The concept of ICU humanization is broad and should involve much more than extended or 24-hour visitation. Measures aimed at patient well-being are part of this humanized care.^(8^) Noise and poor sleep quality are among the main complaints of ICU patients and their relatives.^(1,2^) Excessive noise peaks may occur in the ICU setting and interfere with patient sleep.^(9-11^) Our findings suggest that a minority of the evaluated units measure the noise level (14.4%) or perform interventions to minimize it (such as reducing the volume of alarms at night), revealing an opportunity for improvement in the quality of patient care. Ensuring an environment with controlled noise is a simple, low-cost intervention and falls within the concept of humanized and patient-centered care.

The effect of natural light on critically ill patients has been evaluated for at least a decade. The deprivation of exposure to natural light and exposure to artificial light have been related to changes in the circadian rhythm, sleep disorders, and *delirium*.^(3,12^) However, Khon et al. found no effect of the presence of windows and exposure to sunlight on outcomes in critically ill patients.^(13^) Measures such as exposure to natural light, maintenance of the circadian rhythm, and minimization of artificial light exposure at night are part of a bundle for promoting sleep, with satisfactory results.^(14^) We observed that the majority of the units reduced light at night (80.5%), which is also a feasible and inexpensive intervention for promoting sleep in patients.

The Clinical Practice Guidelines for the Prevention and Management of Pain, Agitation/Sedation, Delirium, Immobility, and Sleep Disruption in Adult Patients in the ICU and the Guidelines for Family-Centered Care in the Neonatal, Pediatric, and Adult ICU, both from ACCCM, make recommendations for sleep promotion in the ICU.^(7,15^) The American PADIS guidelines recognize that some measures, especially nonpharmacological measures, are safe and should be implemented as a sleep promotion bundle, including adjustment of the ventilatory mode at night and reduction of noise and light. These same guidelines do not make recommendations on the use of medications to promote sleep, exception that they recommend against propofol to improve sleep in critically ill patients.^(7^) The guidelines for family-centered care in the ICU recognize that sleep deprivation in the ICU has a negative impact not only on the patient but also on the family and recommend that adequate sleep conditions be offered to the relatives of patients in the ICU.^(15^) Nevertheless, in the evaluated sample, only a small portion of units (15.3%) reported having a sleep promotion protocol. There was no difference between private and public units regarding the institution of sleep promotion measures, although the pharmacological protocol was more frequent in private units. Light reduction at night was also more common in private units (p = 0.01), which is a simple measure that does not require financial investment and which should be more encouraged in Brazilian ICUs. Among the study participants, 99% answered that they believed that poor sleep can negatively affect patient recovery and that adequate sleep reduces the incidence of *delirium*. An international survey study with 1,223 participants, in which 84% of the ICUs were located in the US and 1% in Brazil, showed that a small portion of the units had a sleep promotion protocol (32%). As in our study, most participants believed that poor sleep can affect the patient recovery process.^(6^

This study has some limitations. First, the analyzed sample was small. E-mails were sent to the AMIBnet list and to the e-group with a link to the survey. According to the AMIB census conducted in 2016, Brazil has 1,951 establishments with adult ICUs, 77 coronary ICUs, and 50 burn ICUs.^(16^) A total of 190 questionnaires were filled out, but only 118 were included in the analysis. This factor may have caused sampling bias and limited the external validity of our results because the response rate was approximately 5%. Nevertheless, this is the first description of sleep promotion practices in Brazilian ICUs, and the distribution of responses was similar to the distribution of ICUs among Brazilian regions, with representatives from all regions. Second, the survey was based on previous studies, and it was validated only among our researchers using a method similar to Delphi and not previously externally validated. Third, although the questionnaire evaluates practical aspects of sleep promotion and sedation protocols, we did not specifically evaluate the details of the protocols of units that reported having sleep promotion measures. In addition, we did not ask the units about protocols for the prevention and management of *delirium*. We know that analgesia-sedation and delirium protocols are contiguous with each other. Lastly, we did not assess the ventilatory mode setting at night, another measure that can impact sleep improvement.^(17,18^

The implementation of measures to promote sleep in ICUs is challenging and involves the participation of a multidisciplinary team. Sleep promotion bundles include nonpharmacological and pharmacological measures. Among the nonpharmacological measures, a noise reduction program, light reduction, and patient care adjustment are environmental modification actions that are simple to implement, cost little, have shown several benefits to patients, and may be the first measure to be adopted in the development of a sleep promotion program, but we emphasize that such actions require the engagement of the team to become effective.

## CONCLUSION

In Brazil, there is a gap between the perception of health professionals about the impact of sleep in the intensive care unit and the implementation of measures for promoting sleep. The simplest and least expensive measures for promoting sleep could be implemented in a broad multidisciplinary program. Studies with a more robust methodology should be performed to evaluate the actual impact of a sleep promotion protocol on critically ill patients in various outcomes in this population.

## Annex 1

Questionnaire on sleep promotion practices

**Table:**

1. City: _____________________________ State: _______________

2. Hospital name: __________________________________________

3. ICU Name: _____________________________________________

4. Position of the person responsible for filling out the questionnaire:
Physician Coordinator ( ) Staff Physician ( ) On-duty Physician ( ) Nurse Coordinator ( ) Nurse Writer ( )

5. Number of annual admissions:
Up to 200 ( ) 201 - 400 ( ) 401 - 600 ( ) 601 - 800 ( ) >801 ( )

6. ICU type: Clinical ( ) Surgical ( ) Clinical-Surgical ( ) Trauma ( ) Cardiac ( ) Neurological ( ) Burn ( )

7. Number of beds: _______

8. Primary source of funding: Public ( ) Private ( )

9. Nurse:patient ratio: 1:2 ( ) 1:3 - 5 ( ) 1:6 - 8 ( ) 1:9 - 12 ( ) 1: ≥13 ( ) 1:9 - 12 ( ) 1: ≥13 ( )

10. Physician:patient ratio:
Day 1: ≤5 ( ) 1:6 - 10 ( ) 1:11 - 15 ( ) 1: ≥16 ( )
Night 1: ≤5 ( ) 1:6 - 10 ( ) 1:11 - 15 ( ) 1: ≥16 ( )

11. Do you have a staff intensive care physician? Yes ( ) No ( )

12. Type of division between beds:
None ( ) Curtain ( ) Screen ( ) Partition ( ) Single apartments ( )

13. Infrastructure:
Windows in most of the beds: Yes ( ) No ( )
Clocks on the walls of the beds: Yes ( ) No ( )

14. Number of visit periods: 0 ( ) 1 ( ) 2 ( ) 3 ( ) Continuous ( )

15. Permitted visitation time per period: < 30 minutes ( ) 30 - 60 minutes ( ) 61 - 180 minutes ( ) > 180 minutes ( ) 24 hours ( )

16. Is there a nonpharmacological protocol for promoting sleep in your ICU?
Yes ( ) No ( )
16.1 Is there noise measurement in the ICU?
Yes ( ) No ( )
16.2 Is there a protocol for noise reduction?
Yes ( ) No ( )
16.3 Are alarm volumes (monitors, ventilators) adjusted at night?
Yes ( ) No ( )
16.4 Is there a recommendation to reduce light at night?
Yes ( ) No ( )
16.5 Is there a recommendation to minimize the performance of routine tests (laboratory and imaging) at night, excluding urgencies?
Yes ( ) No ( )
16.6 Does the ICU provide ear plugs to minimize noise?
Yes ( ) No ( )
16.7 Does the ICU provide eye masks to minimize light?
Yes ( ) No ( )
16.8 Does the ICU use music to help induce sleep?
Yes ( ) No ( )
16.9 Does the ICU have an early mobilization program?
Yes ( ) No ( )

17. How do you assess the sleep quality of your patients in the ICU?
Patient report ( ) Multiprofessional team report ( ) Complementary exams (polysomnography, BIS, actigraphy) ( ) I do not routinely assess it ( )

18. Regardingsedation and analgesia strategies:
18.1 Which scale is used? RASS ( ) SAS ( ) RAMSAY ( ) Other ( )
18.2 What is the sedation strategy? Target sedation ( ) Daily awakening ( )
18.3 Who is responsible for the adequacy of the protocol?
Physician ( ) Nurse ( ) Physician and Nurse ( )

19. Does the ICU have a pharmacological protocol for promoting sleep?
Yes ( ) No ( )
19.1 Which drugs are used?
Melatonin ( ) Zolpidem ( ) Benzodiazepine ( ) Neuroleptic ( ) Others ( )

20. Do you believe that poor sleep can affect patient recovery?
Strongly agree ( ) Agree ( ) Neutral ( ) Strongly disagree ( ) Strongly disagree ( )

21. Do you consider that a proper sleep cycle reduces the incidence of *delirium*?
Strongly agree ( ) Agree ( ) Neutral ( ) Strongly disagree ( ) Strongly disagree ( )

22. What is the most important measure to improve sleep in the ICU?
22.1 Allow uninterrupted sleep at night ( ) Strongly agree ( ) Agree ( ) Neutral ( ) Strongly disagree ( ) Strongly disagree ( )

22.2 Noise control. ( ) Strongly agree ( ) Agree ( ) Neutral ( ) Strongly disagree ( ) Strongly disagree ( )

22.3 Mobilization of patients during the day so they can rest at night. ( ) Strongly agree ( ) Agree ( ) Neutral ( ) Strongly disagree ( ) Strongly disagree ( )

22.4 Keeping the ICU bright during the day and reducing light at night. ( ) Strongly agree ( ) Agree ( ) Neutral ( ) Strongly disagree ( ) Strongly disagree ( )

22.5 Keeping patients awake during the day. ( ) Strongly agree ( ) Agree ( ) Neutral ( ) Strongly disagree ( ) Strongly disagree ( )

23. What is the approximate percentage of patients in your ICU who received sleep medication in the last 7 days?
0 - 25% ( ) 26 - 50% ( ) 51 - 75% ( ) 76 - 100% ( ) Do not know ( )

24. Would you like to participate in a study on nonpharmacological interventions for promoting sleep in the ICU?
Yes ( ) No ( )
